# Direct Interstitial Decongestion in an Animal Model of Acute-on-Chronic Ischemic Heart Failure

**DOI:** 10.1016/j.jacbts.2021.09.008

**Published:** 2021-11-22

**Authors:** William T. Abraham, Michael Jonas, Ranjeet M. Dongaonkar, Beth Geist, Yukie Ueyama, Kevin Render, Brad Youngblood, William Muir, Robert Hamlin, Carlos L. del Rio

**Affiliations:** a Division of Cardiovascular Medicine, The Ohio State University, Columbus, Ohio, USA; b Department of Cardiology, Kaplan Medical Center, Hebrew University School of Medicine, Rehovot, Israel; c Department of Veterinary Physiology & Pharmacology, Michael E. DeBakey Institute for Comparative Cardiovascular Science and Biomedical Devices, Texas A&M University, College Station, Texas, USA; d QTest Labs, Columbus, Ohio, USA; e Cardiac Consulting, San Mateo, California, USA

**Keywords:** acute decompensated heart failure, decongestion, interstitial, lymphatic, pulmonary edema, thoracic duct, volume overload, ADHF, acute decompensated heart failure, CVP, central venous pressure, EVLW, extravascular lung water

## Abstract

Removal of excess fluid in acute decompensated heart failure (ADHF) targets the intravascular space, whereas most fluid resides in the interstitial space. The authors evaluated an approach to interstitial decongestion using a device to enhance lymph flow. The device was deployed in sheep with induced heart failure (HF) and acute volume overload to create a low-pressure zone at the thoracic duct outlet. Treatment decreased extravascular lung water (EVLW) volume (mL/kg) (-32% ± 9%, *P* = 0.029) compared to controls (+46% ± 9%, *P* = 0.003). Device-mediated thoracic duct decompression effectively reduced EVLW. Human studies may establish device-based interstitial decongestion as a new ADHF treatment.

## Highlights

• In ADHF, elevated CVP opposes thoracic duct lymph flow and impairs decongestion of the interstitial space.
• The use of a novel device for reducing CVP at the outflow of the thoracic duct was shown to be safe, well-tolerated, and effectively reduced EVLW, in an animal model of acute-on-chronic ischemic HF.
• Similar results were observed when translating this therapy to a human case study.
• Additional human studies to confirm these findings may establish device-based direct interstitial decongestion as a new treatment for ADHF.

## Visual Abstract

**Figure:**
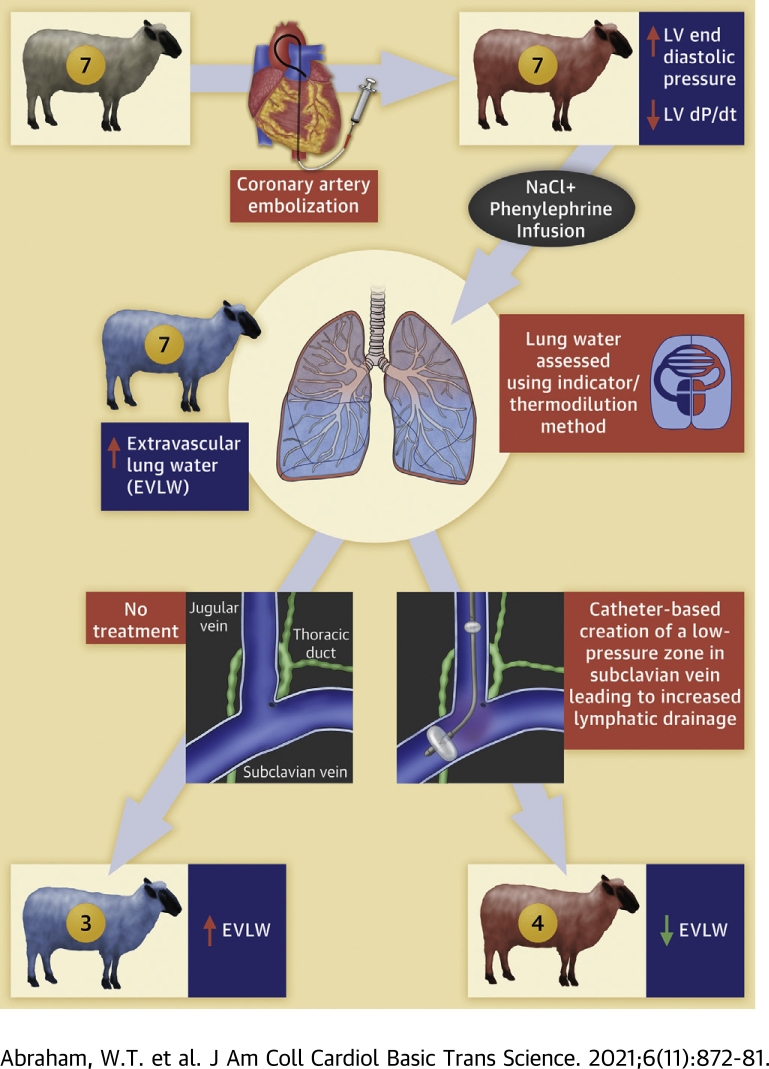

Acute decompensated heart failure (ADHF) is the primary cause of more than 1 million hospitalizations and plays a role in an additional 2 million hospitalizations annually in the United States (1). Extracellular fluid volume excess, manifesting clinically as worsening shortness of breath and peripheral edema, is present in approximately 90% of patients at the time of admission for ADHF and often referred to as clinical congestion. Thus, alleviating clinical congestion and its associated symptoms is one of the main targets to improve clinical outcomes during ADHF therapy. Inadequate decongestion resulting in residual congestion at the time of discharge is a strong predictor of poor post-discharge outcomes and readmission (2, 3, 4, 5).

Current decongestive strategies for the treatment of ADHF primarily target removal of fluid from the intravascular space using diuretic or less frequently ultrafiltration therapies, even though the majority of extracellular fluid volume excess resides within the interstitial space in these patients. Lymphatic drainage is the primary route for removal of excess interstitial fluid. However, early on with the current approach, the elevated central venous pressure (CVP) associated with ADHF opposes thoracic duct lymph flow and interstitial decongestion remains impaired (6,7). Subsequently, after a significant reduction of intravascular fluid volume and CVP, interstitial decongestion occurs passively and slowly, and is often insufficient leading to residual congestion. Unfortunately, this sequence of events is inefficient and may result in relative underfilling of the intravascular space despite an ongoing excess of total body fluid volume leading to hypotension and worsening prerenal azotemia, thus limiting treatment.

An approach that targets simultaneous rather than sequential fluid removal from the intravascular and interstitial spaces may prove more efficacious and avoid the limitations of current therapy in ADHF patients. Since reducing the outflow pressure at the thoracic duct has been shown to enhance lymph flow, a device-based approach (WhiteSwell System, WhiteSwell) was designed to create a low-pressure zone in the outflow area of the thoracic duct to promote increased lymph flow, thus draining fluid from the interstitial space into the intravascular space, with simultaneous removal of excess fluid from the intravascular space using diuretics (6, 7, 8). The present study was designed to evaluate the safety and potential effectiveness of this device in animals with acute on chronic ischemic heart failure (HF), thus providing proof of concept. In addition, a single human case is reported to demonstrate the feasibility to translate this approach to clinical care.

## Methods

### Experimental animals

This study was performed using 7 sheep (54.3 ± 3.9 kg) obtained from the Ohio State University Sheep Center and housed and studied at QTest Labs. One additional healthy sheep was used to pilot and troubleshoot the device deployment/operation before the study of these 7 animals and had an uneventful course. The study protocol was approved by QTest Labs’ Institutional Animal Care and Use Committee for compliance with regulations and current acceptable practices. The animals were individually housed in stainless steel runs during the entire in-life phase of this study. The cages conformed to the standards set forth in the Guide for the Care and Use of Laboratory Animals: Eighth Edition and the current USDA Animal Welfare Act as amended (9,10). Each run was clearly labeled with a card that, at a minimum, displayed the study number, the sheep's identification number, and sex. The environmental conditions of the animal room during the study conformed to the following: 1) the light/dark cycle was set to maintain approximately 12 hours of light and 12 hours of dark each day; 2) the room temperature and relative humidity controls were set to maintain temperatures of 61°F to 81°F (16°C to 27°C), and relative humidity between 30% and 70%. Given the gregarious nature of sheep, all sheep were housed in a manner supporting auditory, visual, and olfactory communication with neighboring sheep.

### Investigational device

**Figure 1:**
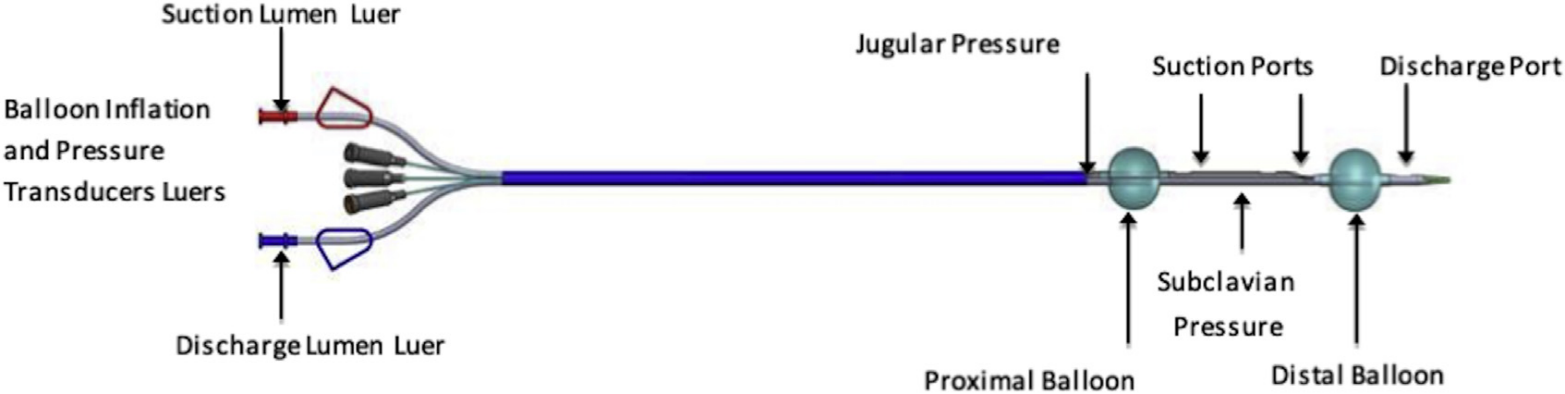
First-Generation Whiteswell Catheter See text for description.

The WhiteSwell device consisted of a multilumen catheter with 2 compliant balloons made of low-durometer urethane (1 proximal and 1 distal) (Figure 1), as well as a blood-pump mechanism that allowed the withdrawal of blood in between balloons and return of blood via the distal end. This system was designed to decrease the local pressure in the area encompassed between the balloons while preserving distal blood flow. In short, the WhiteSwell System seeks to locally reduce venous pressures in the thoracic duct outflow area to facilitate interstitial fluid drainage while preserving left internal jugular and left subclavian flow. For this purpose, a catheter with 2 spaced-apart balloons was positioned across the bifurcation of the jugular and innominate veins, the balloons when inflated isolating the thoracic duct outflow. Both balloons had built in flow paths that facilitate jugular and subclavian flow and a blood pump lowered the pressure in the isolated region.

The system also allows the continuous monitoring of pressures both in between the balloons (i.e., thoracic duct outflow/subclavian) as well as at a proximal (i.e., jugular) location. Pressure measurements for pump control are performed by 2 external pressure transducers, which are connected to 2 small tubes which end at different locations along the catheter.

### Study design

The sheep underwent a well-established serial coronary artery embolization procedure to trigger left ventricular dysfunction and pathological remodeling consistent with a chronic HF syndrome. Over the course of the embolization protocol, left ventricular dilation and systolic dysfunction (i.e., reduced ejection fraction <30%) were confirmed by echocardiography.

Following the induction of chronic systolic HF, the sheep were anesthetized and instrumented to permit the collection of electrocardiogram, hemodynamic variables (cardiac output, systemic [arterial], right atrial/central venous, and left ventricular pressures), urine output, and blood samples. Animals were also instrumented for the determination of intravascular and total lung water volume using dual indicator/thermodilution method. Treatment animals (n = 4) had the WhiteSwell device deployed via percutaneous access to the left jugular vein; to preserve patency, the device was routinely/continuously flushed with a low-volume heparinized solution. Fluoroscopy associated with a contrast injection was used to place the distal balloon within the cranial vena cava and the proximal balloon within the left jugular vein.

Following instrumentation and hemodynamic stabilization, baseline data (hemodynamic, lung water volume, urine output, and blood sampling) were collected. Then, acute cardiac decompensation with pulmonary congestion was induced by a combination of fluid overload using sodium chloride (NaCl) 0.9% or other suitable crystalloid at up to 90 mL/kg intravenous bolus and followed by 1 to 2 L/h intravenous and pressure overload using phenylephrine 1 to 10 μg/kg/min. Cardiac congestion was defined as a CVP >10 mm Hg and a left ventricular end-diastolic pressure >23 mm Hg, whereas pulmonary edema was defined as a significant increase (50% or 100 mL from normal values) in lung water volume; edema was confirmed by dual indicator dilution method using cooled saline and hetastarch injected into the right atrium and measured at the level of the pulmonary artery and ascending aorta. Once a steady state for lung water volume had been achieved for up to 1 hour, the WhiteSwell device was activated for up to 3 hours in the 4 treated animals; 3 animals served as controls. Subsequently, the animals were euthanatized.

### Statistical analysis

Descriptive statistics were used to summarize all data collected during the study (e.g., pre- and postinduction of ischemic HF). Continuous variables are presented as means with standard error of the means (mean ± SEM). Comparisons between healthy sheep (control, n = 7, with 6 historical) and those with induced ischemic HF (n = 7) were made using unpaired Student *t* tests. Differences before/after induction of ADHF (in all HF sheep) as well as comparisons within treatment arms (i.e., before/after each treatment) were evaluated using paired Student *t* tests. Normality assumptions were verified via the Shapiro-Wilk test; if the data were not normally distributed, then rank differences were evaluated via either the Mann-Whitney (unpaired comparisons) or the Wilcoxon test (paired comparisons). Device efficacy was further evaluated by studying the rate of extravascular lung water (EVLW) accumulation during the treatment period via least-squares linear regression (slope of EVLW vs. time on treatment), both independently for each animal and as group. Results are presented using derived slopes and the square of Pearson's correlation coefficient (R^2^), whereas group-fitted models (slopes) were compared via the Akaike Information Criterion. Individual EVLW accumulation slopes and changes before/after treatment among groups were also further compared via independent tests (as described above). In all instances, statistical analyses were performed using Prism (version 9; GraphPad Software, LLC), and a 2-sided (when pertinent) *P* of 0.05 or less was considered (a priori) as evidence of statistical significance.

## Results

### Induction of chronic and acute on chronic HF

Induction of chronic systolic HF was successfully performed in all 7 animals. These animals underwent up to 2 separate serial coronary embolization procedures and were subsequently (26 ± 6 days later) terminally anesthetized/instrumented for the acute study. Embolized sheep exhibited hemodynamic and mechanical indices consistent with chronic left ventricular dysfunction and HF (Table 1). The isoflurane-anesthetized embolized sheep had elevated left ventricular filling pressures (end-diastolic pressure [EDP], 16.4 ± 1.4 mm Hg), depressed indices of systolic function (dP/dt_40_, 1,183 ± 5 mm Hg/s; and contractility index 29.6±0.1 1/s), slowed relaxation (e.g., tau, 25.5 ± 3.6 ms), and elevated CVPs (5.8 ± 0.4 mm Hg), compared to 6 historical healthy controls and the 1 healthy sheep included in this study (Table 1). Chronic HF sheep also had detectable basal levels of EVLW (202 ± 20 mL or 3.9 ± 0.5 mL/kg). For comparison, the basal EVLW in the healthy sheep was 31 mL. Taken together, these data confirm an induced pathophysiology consistent with the clinical manifestations of the HF phenotype.

**Table 1:** Hemodynamic Variables and Extravascular Lung Water Content Following Induction of Chronic Ischemic Heart Failure

	Heart Failure (n = 7)	Healthy (n = 7)	*P* Value
HR, beats/min	67 ± 2	117 ± 6	<0.001
MSP, mm Hg	81 ± 5	79 ± 9	0.862
CVP, mm Hg^a^	5.8 ± 0.4	2.2 ± 0.9	0.004
EDP, mm Hg^b^	16.4 ± 1.4	3.9 ± 1.4	<0.001
+dP/dt_40_, mm Hg/s^b^	1,183 ± 5	1,684 ± 118	0.001
-dP/dt_40_, mm Hg/s^b^	-759 ± 67	-1,320 ± 160	0.007
Contractility index, 1/s^b^	29.6 ± 0.1	42.1 ± 2.9	0.001
LV-tau, ms^b^	25.5 ± 3.6	16.6 ± 1.8	<0.05
EVLW, mL^c^	202 ± 20	31 (n = 1)	***—***

Values are mean ± SEM; healthy animal data taken from both historical controls (n = 6) and 1 healthy animal studied under this protocol.

CVP = central venous pressure; dP/dt_40_ = the first derivative of the left ventricular pressure signal at a developed left ventricular pressure of 40 mm Hg; EDP = end-diastolic pressure; EVLW = extravascular lung water HR = heart rate; LV-Tau = left ventricular–tau; MSP = mean systolic pressure.

a Derived/estimated from the mean right-atrial pressure.

b Derived/estimated from the left-ventricular pressure signal, where contractility index = +dP/dt_40_/40 mm Hg.

c Derived/measured via dual-indicator thermodilution (5 mL of either cold 0.9% NaCl or cold 6% hetastarch solutions) using a right-sided Swan-Ganz catheter advanced into the pulmonary artery trunk, and a thermistor catheter advanced into the aortic arch.

The combination of both fluid (2.0 ± 0.2 L/h with a 1.3 ± 0.2 L/h loading bolus of crystalloid) and pressure (11.9 ± 1.0 mg/h or 3.8 ± 0.6 μg/kg/min of phenylephrine) overload triggered immediate and sustained increase in intravascular lung water volume consistent with congestion, and progressive increase in EVLW volume in all animals. After 94.3 ± 4.3 minutes of overload, EVLW volume increased 119 ± 20% from baseline (202 ± 20 mL vs. 432.4 ± 45.3 mL; *P* = 0.001) and, despite sustained ventilation (100% fraction of inspired oxygen), arterial oxygen tension (partial pressure of oxygen [PaO_2_]) decreased 23% ± 9% below baseline levels (487 ± 9 mm Hg vs. 372 ± 40 mm Hg; *P* = 0.039), consistent with pulmonary edema and acute decompensation of HF (Table 2). In addition, overload had negligible effects on cardiac function but markedly increased filling (EDP, +107 ± 18% to 32.8 ± 1.3 mm Hg; *P* = 0.016 vs. baseline) and CVPs (+111 ± 13% to 12.0 ± 0.8 mm Hg; *P* < 0.001 vs. baseline), resulting in an elevated transpulmonary gradient (10.7 ± 1.5 mm Hg to 20.8 ± 1.6 mm Hg; *P* = 0.002). During the acute decompensation period, EVLW accumulated at a rate of 2.45 ± 0.45 mL/min (R^2^ = 0.97 ± 0.02; *P* < 0.001), whereas PaO_2_ decreased (-1.34 ± 0.41 mm Hg/min, R^2^ = 0.85 ± 0.08; *P* = 0.002).

**Table 2:** Hemodynamic Variables, Extravascular Lung Water Content, and Other Parameters Following Induction of Acute on Chronic Ischemic Heart Failure (N = 7)

	Baseline (HF)	ADHF	*P* Value
HR, beats/min	67 ± 2	79 ± 9	0.247
MSP, mm Hg	81 ± 5	146 ± 7	<0.001
CVP, mm Hg^a^	5.8 ± 0.4	12.0 ± 0.8	<0.001
EDP, mm Hg^b^	16.4 ± 1.4	32.8 ± 1.3	0.016
+dP/dt_40_, mm Hg/s^b^	1,183 ± 5	1,116 ± 88	0.471
-dP/dt_40_, mm Hg/s^b^	-759 ± 67	-938 ± 114	0.063
Contractility index, 1/s^b^	29.6 ± 0.1	27.9 ± 2.2	0.470
EVLW, mL^c^	202 ± 20	432 ± 45	0.001
EVLW, mL/kg^c^	3.9 ± 0.5	8.1 ± 0.7	<0.001
IVLW, mL^c^	350 ± 50	451 ± 57	0.013
PaO_2_, mm Hg	487 ± 9	372 ± 40	0.039
Cardiac output, L/min^c^	8.4 ± 1.0	11.5 ± 1.0	0.010
Plasma hemoglobin, g/L	0.39 ± 0.07	0.61 ± 0.12	0.007

Values are mean ± SEM; ADHF induced via the overlapping combination of both fluid and pressure overload; data are reported after 94.3±4.3 minutes of overload.

ADHF = acute decompensated heart failure; HF = heart failure; IVLW = intravascular lung water; PaO_2 =_ partial pressure of oxygen; other abbreviations as in Table 1.

a Derived/estimated from the mean right-atrial pressure.

b Derived/estimated from the left-ventricular pressure signal, where contractility index = +dP/dt_40_/40 mm Hg.

c Derived/measured via dual-indicator thermodilution (5 mL of either cold 0.9% NaCl or cold 6% hetastarch solutions) using a right-sided Swan-Ganz catheter advanced into the pulmonary-artery trunk, and a thermistor catheter advanced into the aortic arch.

### Response to WhiteSwell therapy

The WhiteSwell device was successfully deployed and operated in 4 acutely decompensated HF sheep and the responses were compared to time-matched responses in 3 acutely decompensated HF animals without the device. The WhiteSwell device was well tolerated (both when operational and/or deactivated) in all treated animals; no evidence of arrhythmic, hemodynamic, and/or mechanical adverse events was observed. Upon activation, the device successfully created a low-pressure zone at the outlet of the thoracic duct. The average pressure measured between the 2 balloons in the low-pressure zone was ∼2 mm Hg (range: -3 to 5.7 mm Hg), whereas the device-recorded CVP was 12.0 ± 0.8 mm Hg (vs. 11.8 ± 1.2 mm Hg recorded with a fluid-filled transduced/catheter).

**Figure 2:**
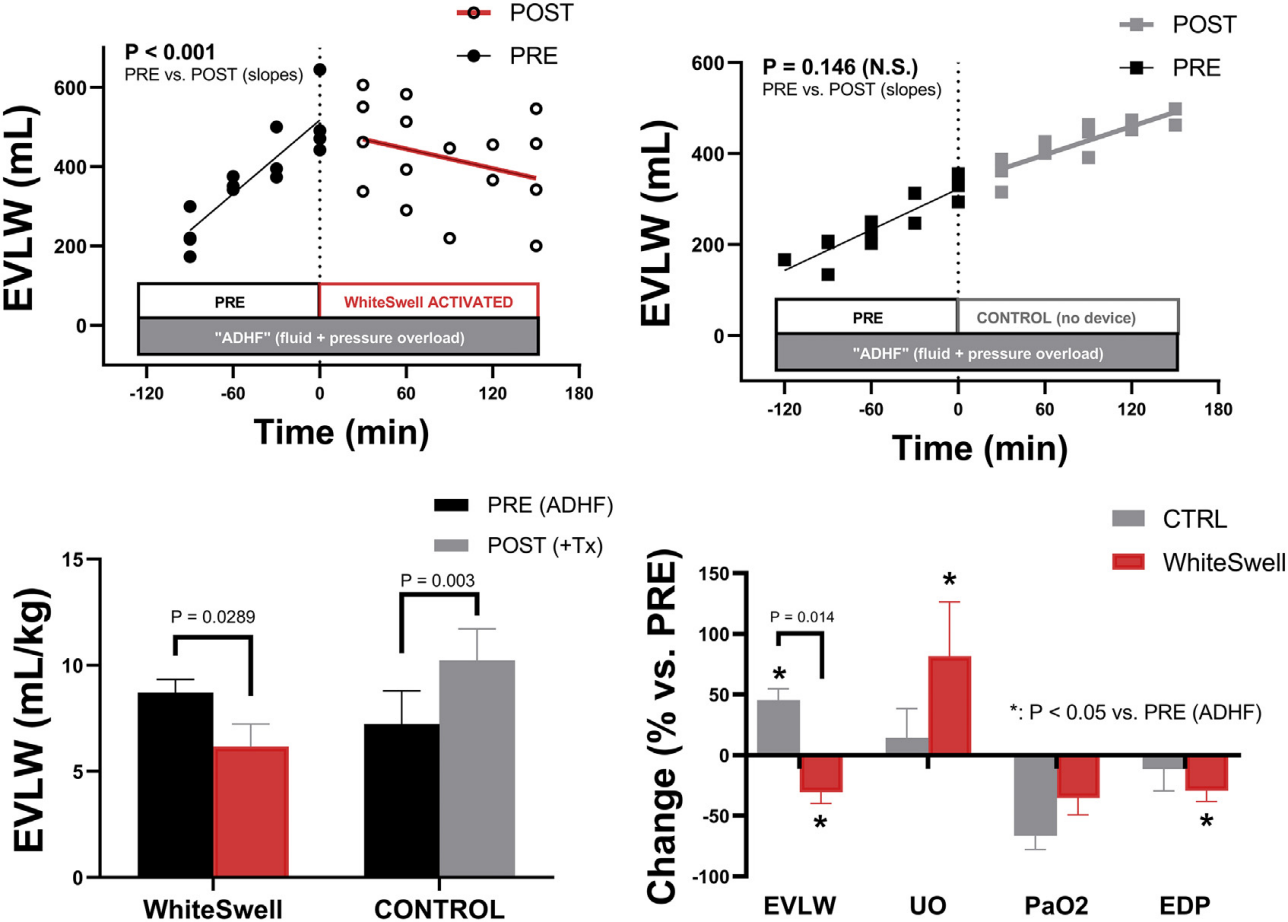
Extravascular Lung Water Changes **(Top)** Changes in the estimated extravascular lung water content (EVLW) as measured in sheep with induced acute decompensation (ADHF) both before and during treatment with the WhiteSwell device **(left)**. Changes in time-matched untreated controls (CTRL) are shown **(right)** for comparison. **(Bottom)** Absolute and/or relative changes post-treatment in EVLW (mL/kg), arterial oxygen tension (PaO_2_), left-ventricular filling pressures (EDP), and cardiac output (CO).

Activation of the device resulted in marked beneficial cardiopulmonary changes, primarily in EVLW and oxygenation (Figure 2). EVLW decreased significantly in animals treated with the device (Figure 2, Table 3) (8.7 ± 0.6 mL/kg to 6.2 ± 1.1 mL/kg or 512 ± 45 mL to 358 ± 58 mL; *P* = 0.029 and *P* = 0.038, respectively), whereas EVLW continued to increase in the untreated time-matched controls (Figure 2, Table 3) (7.2 ± 1.6 mL/kg to 10.2 ± 1.5 mL/kg or 326 ± 18 mL to 471 ± 14 mL; *P* = 0.003 and *P* = 0.025, respectively). These changes in EVLW between groups were statistically different at the end of the study (*P* = 0.014). In other words, when compared against untreated animals, activation of the WhiteSwell device not only abolished further EVLW/edema accumulation, but effectively decreased it. Indeed, during the treatment period, EVLW accumulated at a rate of 1.05 ± 0.14 mL/min (R^2^ = 0.92 ± 0.07) in controls but decreased -0.82 ± 0.15 mL/min in treated animals (R^2^ = 0.84 ± 0.04) (*P* = 0.006 for treated vs. control).

**Table 3:** Changes in Hemodynamics, Extravascular Lung Water Content, and in Treated and Control Animals

	ADHF Treatment Group (n = 4)			ADHF Control Group (n = 3)		
	ADHF	ADHF + Treatment	*P* < 0.05^a^	ADHF	ADHF + Control	*P* < 0.05^a^
HR, beats/min	73 ± 9	94 ± 6	—	86 ± 19	111 ± 4	—
MSP, mm Hg	142 ± 8	116 ± 17	—	153 ± 15	117 ± 10	0.026 (↓)
CVP, mm Hg^b^	11.8 ± 0.8	10.7 ± 1.0	—	12.4 ± 1.9	15.0 ± 4.7	—
EDP, mm Hg^c^	34.7 ± 0.2	24.5 ± 3.0	0.038 (↓)	30.2 ± 2.3	27.5 ± 7.6	—
+dP/dt_40_, mm Hg/s^c^	1,044 ± 117	1,452 ± 301	—	1,213 ± 137	1,306 ± 40	—
-dP/dt_40_, mm Hg/s^c^	-800 ± 124	-724 ± 108	—	-1,121 ± 176	-773 ± 44	—
Contractility index, 1/s^c^	26.1 ± 2.9	36.3 ± 7.5	—	30.3 ± 3.4	21.8 ± 10.9	—
EVLW, mL^d^	512 ± 45	358 ± 58	0.038 (↓)	326 ± 18	471 ± 14	—
EVLW, mL/kg^d^	8.7 ± 0.6	6.2 ± 1.1	0.029 (↓)	7.2 ± 1.6	10.2 ± 1.5	0.003 (↑)
IVLW, mL^d^	476 ± 87	396 ± 64	—	417 ± 80	446 ± 109	—
PaO_2_, mm Hg	408 ± 27	273 ± 68	—	325 ± 88	101 ± 48	—
Cardiac output, L/min^d^	11.8 ± 1.2	10.2 ± 1.5	0.034 (↓)	11.1 ± 1.9	8.1 ± 0.5	—
Urine output, mL, %input^e^	1,169 ± 419 (22 ± 6)	1,932 ± 417 (33 ± 5)	0.043 (↑)	729 ± 243 (22 ± 7)	1,132 ± 193 (23 ± 4)	—
Plasma hemoglobin, g/L	0.48 ± 0.11	0.63 ± 0.20	—	0.80 ± 0.20	1.10 ± 0.15	—

Values are mean ± SEM; ADHF induced via the overlapping combination of both fluid and pressure overload; data are reported after 94.3 ± 4.3 min of overload.

Abbreviations as in Tables 1 and 2.

a Versus ADHF, reflecting either an increase (↑) or decrease (↓).

b Derived/estimated from the mean right-atrial pressure (RAP).

c Derived/estimated from the left-ventricular pressure signal, where CI = +dP/dt_40_/40 mmHg.

d Derived/measured via dual-indicator thermo-dilution (5 mL of either cold 0.9% NaCl or cold 6% hetastarch solutions) using a right-sided Swan-Ganz catheter advanced into the pulmonary-artery trunk, and a thermistor catheter advanced into the aortic arch.

e Total urine output as recorded during ADHF induction, and during the treatment period, with the percentages of the respective total fluid input shown in parenthesis.

Although oxygenation continued to deteriorate during the treatment/control period in all animals, treatment with the device tended to blunt PaO_2_ decreases (-35% ± 14% to 273 ± 68 mm Hg in treated animals vs. -66% ± 11%, to 101 ± 48 mm Hg in controls; *P* = 0.137 treated vs. untreated), consistent with the observed changes in EVLW volume. For animals with adequate oxygenation (i.e., PaO_2_ > 350 mm Hg) before the onset of therapy (3 treated and 2 controls), all untreated controls reached PaO_2_ values below 200 mm Hg at the end of the study (403 to 43 mm Hg and 423 to 196 mm Hg), whereas no treated animal approached that threshold (98 to 306 mm Hg, 458 to 418 mm Hg, and 438 to 280 mm Hg).

Negligible differences in systemic hemodynamics were noted among groups during the treatment period (Table 3). However, moderately lower left ventricular filling pressures were noted in treated animals at the end of the study (EDP 30% ± 9% or 34.7 ± 0.2 mm Hg to 24.5 ± 3.0 mm Hg; *P* = 0.038), resulting in decreased transpulmonary gradient (-41% ± 8% or 23.0 ± 0.9 mm Hg to 13.8 ± 2.2 mm Hg; *P* = 0.015). In addition, both absolute (*P* = 0.043) and relative (to the fluid intake; *P* = 0.091) urine output tended to increase only in treated animals (Table 3). These effects were not detectable in untreated animals; however, intergroup comparisons did not reach statistical significance.

Finally, no evidence of hemolysis was observed with device therapy, as comparable changes in plasma hemoglobin concentrations were noted both in the treatment and control groups (Table 3).

### Translation to humans

**Figure 3:**
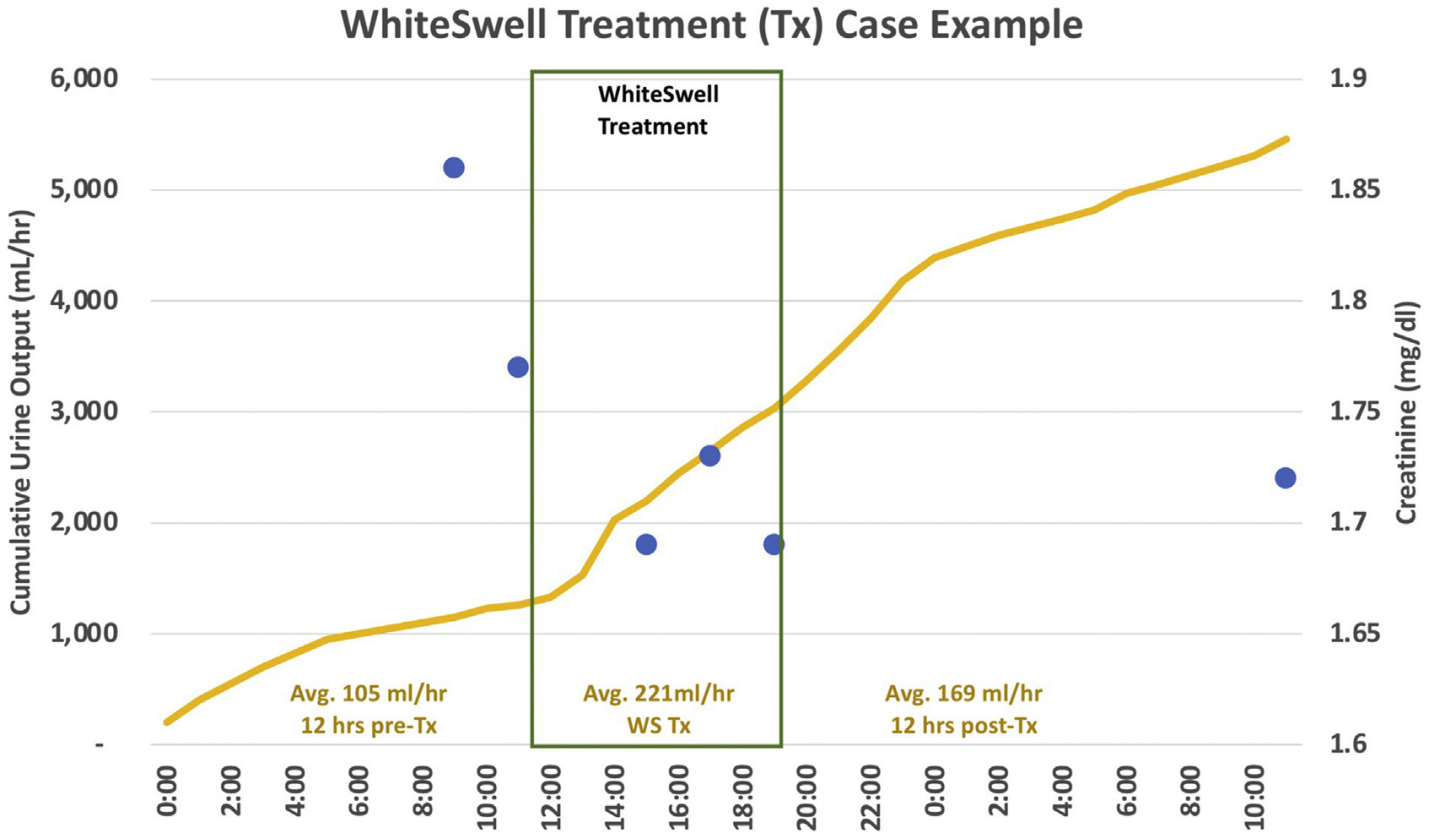
Clinical Case Example With Whiteswell Treatment During WhiteSwell treatment, urine output increased **(yellow)**. Comparing post-therapy to pretreatment, the central venous pressure decreased from 17 mm Hg to 3 mm Hg, and creatinine levels fell slightly **(blue dots)**.

An ongoing U.S. Food and Drug Administration Early Feasibility study (NCT02863796) is evaluating this approach to ADHF treatment in humans. The results from a single subject are presented in Figure 3. The patient was an 82-year-old woman with heart failure with preserved ejection fraction and with hypertension, chronic atrial fibrillation with novel oral anticoagulants, chronic renal failure, and severe pulmonary hypertension. The patient presented to the emergency department with severe right and left heart failure, dyspnea, orthopnea, paroxysmal nocturnal dyspnea, and edema. Intravenous diuretics were administered 2 days before WhiteSwell treatment and continued at the same dose during treatment. The WhiteSwell device was introduced via the left internal jugular vein in a 30-minute catheter lab procedure under fluoroscopy, and a low-pressure zone at the thoracic duct region was achieved and maintained for a treatment period of several hours. Urine output rate increased significantly during WhiteSwell therapy with no change in diuretic dose, while creatinine was reduced. CVP was reduced from 17 mm Hg to 3 mm Hg. N-terminal pro–B-type natriuretic peptide was 12,703 pg/mL pretreatment and 9,025 pg/mL post-treatment. After WhiteSwell therapy, the patient was feeling well with reduced orthopnea and edema.

**Figure 4:**
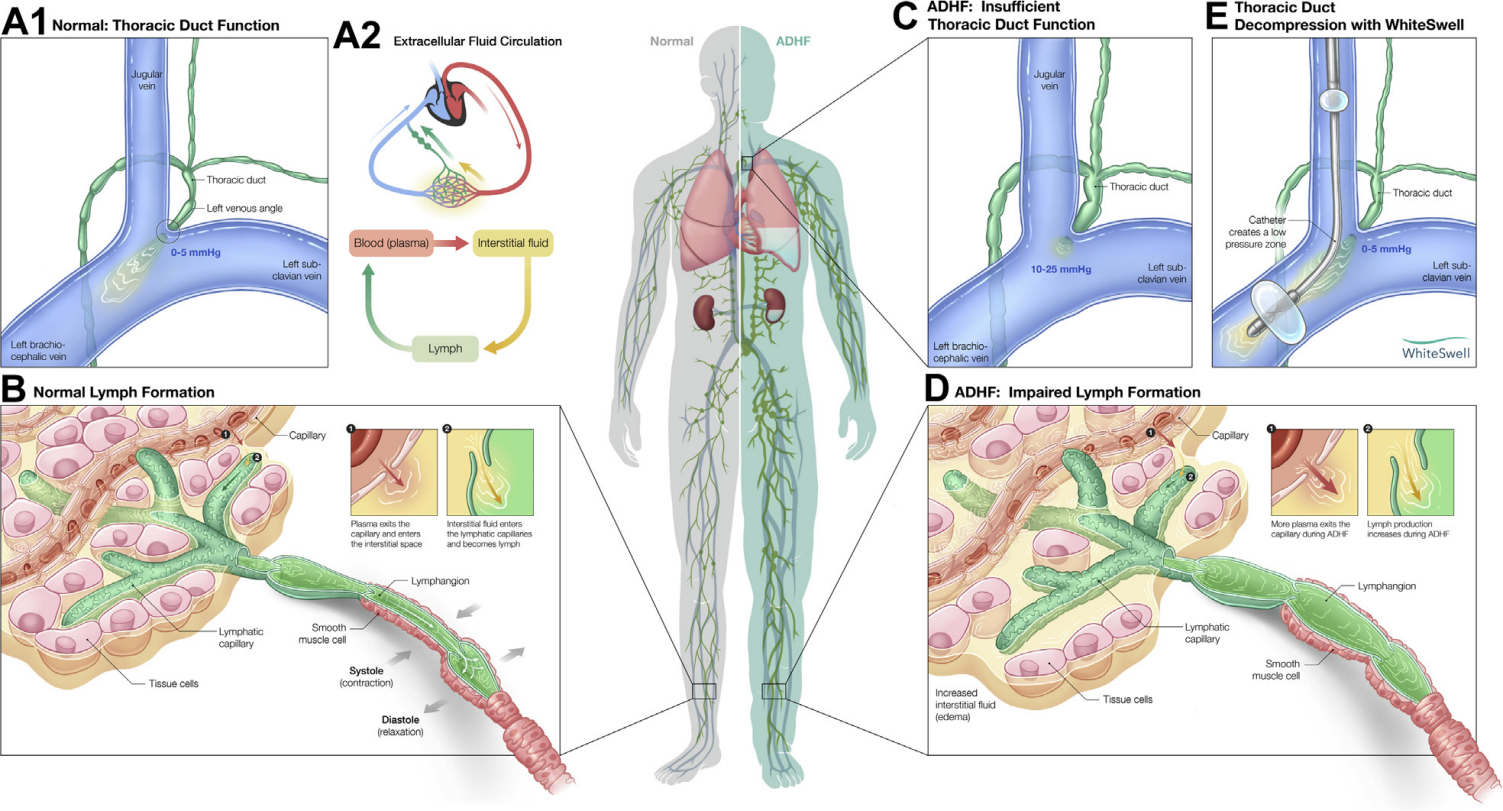
Lymphatic System and Thoracic Duct Function in Healthy Individuals and in Acute Decompensated Heart Failure **(A and B)** Normal thoracic duct function and lymph formation. **(C and D)** Pathological changes in thoracic duct function and lymph formation in the setting of acute decompensated heart failure (ADHF). **(E)** Thoracic duct decompression and restoration of lymph flow during treatment with the WhiteSwell device. See text for detailed explanations. Figure by Avesta Rastan.

## Discussion

In this large animal model of acute on chronic HF, deployment and activation of a device for reducing pressure at the outflow of the thoracic duct was safe, well-tolerated, and effectively reduced EVLW, thus providing proof-of-concept supporting this novel approach to the treatment of ADHF. The device performed as intended by creating a physiologically normal low-pressure zone of approximately 2 mm Hg at the level of the thoracic duct, compared to the elevated systemic venous pressure of approximately 12 mm Hg in this model of ADHF, resulting in the observed reduction in EVLW. This approach of direct interstitial decongestion targets a major but often neglected system of fluid removal, namely the lymphatic system, and promotes simultaneous rather than sequential removal of fluid excess from the intravascular and extravascular compartments.

Fluid and protein filter from the microvasculature into the interstitium and are removed from the interstitium via lymph flow through the lymphatic system, as detailed in Figure 4. Lymph (i.e., interstitial fluid in a lymphatic vessel) is actively transported from the interstitium by intrinsic and/or extrinsic pumping mechanisms and drained into the venous circulation. The thoracic duct returns lymph from approximately 75% of the body into the venous system near the junction of the left internal jugular and left subclavian veins. Because lymph from the thoracic duct flows into the venous system against CVP, the normal low levels of CVP between 0 and 5 mm Hg are critical for maintaining thoracic duct lymph flow (11, 12, 13, 14, 15).

The balance between the microvascular filtration rate and lymphatic drainage rate determines interstitial fluid volume, making lymphatic system function critical for regulation of extravascular fluid volume. However, perturbations leading to microvascular fluid filtration rate higher than lymph flow rate induce excess interstitial fluid accumulation and edema formation.

In the setting of ADHF, the lymphatic system may be structurally normal, but functionally impaired. On one hand, elevated CVP and vascular congestion associated with ADHF lead to increased microvascular fluid filtration into the interstitial compartment at a rate higher than the rate of drainage by the lymphatic vessels. Concomitantly, drainage of lymph from the thoracic duct into the venous circulation is hindered by the elevated CVP (generally in the range of 10 to 25 mm Hg). The elevated CVP-mediated decrease in thoracic duct lymph flow results in engorgement of upstream lymphatic vessels, and the built-up back-pressure decreases lymph flow further increasing interstitial fluid volume independently of increases in microvascular filtration. Clinically, the impaired lymphatic system function in ADHF contributes to overt interstitial fluid overload, edema formation, and compromised organ function. By creating a localized low-pressure zone at the thoracic duct outflow, the WhiteSwell device helps to increase thoracic duct lymph flow and restore normal function of the upstream lymphatic vessels, enabling removal of fluid from the interstitial compartment into the vascular compartment even when the CVP remains elevated.

This WhiteSwell device-based approach to the treatment of ADHF has 3 critical advantages over the current approaches. First, enhanced thoracic duct lymph flow and restored upstream lymphatic function may significantly hasten interstitial decongestion, resulting in earlier improvement in symptoms and faster recovery of organ function. Second, locally decreased thoracic duct outflow pressure by isolating the thoracic duct outlet from rest of the venous circulation may enable interstitial decongestion to begin even when CVP is significantly elevated at the onset of diuretic therapy. Third, enabling direct interstitial decongestion independent of the levels of CVP may eliminate the need for extreme or aggressive diuresis required to lower CVP and subsequently initiate interstitial decongestion. As a result, avoiding intravascular volume depletion (so-called intravascular or arterial underfilling) and keeping the vascular space full by limiting diuresis may lessen the risk of hypotension and worsening prerenal azotemia that frequently limits current ADHF therapy.

A potential limitation of the preclinical study is that lymph flow was not directly measured; however, the design of this study was to prove the concept that EVLW accumulation could be attenuated with WhiteSwell therapy, and this effect was shown using validated measures of EVLW. Future studies may benefit from more direct measures of lymph flow. The assessment of hemolysis using the measurement of plasma hemoglobin may also be somewhat limited, and future studies may use more comprehensive hemolysis assessments. In addition, the preclinical study did not evaluate the effects of the device on top of guideline-directed medical therapy for HF or in heart failure with preserved ejection fraction. Such considerations represent future directions in the preclinical and clinical development of WhiteSwell Therapy.

Finally, the feasibility of translating this approach to humans has been shown in an ongoing early feasibility study, exemplified by the case presented here. Whereas this first-generation device technology has allowed early assessment of proof-of-concept in both animals and in humans, the next iteration of this device integrates an endovascular pumping system that promises to simplify deployment of the device as well as the clinical management of patients with ADHF. Further human studies to confirm these findings are critical to establish device-based direct interstitial decongestion as a new treatment for acute decompensated HF.

Perspectives **COMPETENCY IN MEDICAL KNOWLEDGE:** The lymphatic system plays a critical role in transferring fluid from the interstitial space back to the vascular compartment, but can be functionally impaired in a setting of ADHF and elevated CVP. Creation of a low-pressure zone at the thoracic duct outflow may enhance lymph flow, moving fluid from the interstitial to the intravascular compartment. Deployment and activation of a device for reducing pressure at the outflow of the thoracic duct was safe, well-tolerated, and effectively reduced EVLW volume.

**TRANSLATIONAL OUTLOOK:** The first generation of the WhiteSwell system has been tested in preclinical models and also in early feasibility clinical studies, showing proof-of-concept. The next iteration of this device which integrates an endovascular pumping system for simplified deployment and clinical management of patients has been tested in preclinical models and will be evaluated in human studies. Further human studies to confirm these findings are critical to establish device-based direct interstitial decongestion as a new treatment for acute decompensated HF.

## Funding Support and Author Disclosures

WhiteSwell funded the studies. Drs Abraham, Jonas, and Dongaonkar have received consulting fees from WhiteSwell. Drs Geist, Ueyama, Render, Youngblood, Muir, Hamlin, and del Rio have received research fees from WhiteSwell for the conduct of the animal studies.
